# A case report of high cervical spinal infarction after stenting with severe stenosis at the beginning of left vertebral artery

**DOI:** 10.1097/MD.0000000000039161

**Published:** 2024-08-09

**Authors:** Wen Cheng, Jiangbin Wu, Quanlong Yang, Xiaodong Yuan

**Affiliations:** a Department of Neurosurgery, Affiliated Hospital and Clinical Medical College of Chengdu University, Chengdu, Sichuan, China.

**Keywords:** case report, complications, literature review, spinal cord infarction, vertebral artery stenting

## Abstract

**Background:**

Spinal cord infarction is an uncommon nervous system disorder. We present a case of high cervical cord infarction caused by stenting of the origin of the left vertebral artery (VA). The incidence of spinal cord infarction is minimal, and it must be distinguished from a number of other disorders. The diagnosis is primarily based on imaging, clinical symptoms, and history. Currently, there is no focused treatment for spinal cord infarction. Thrombolysis, high-dose glucocorticoid shocks, tube dilatation to promote circulation, and nutritional neurotropic medicines given early in the course of the disease can all help to slow the disease’s progression. There is no agreement on the etiology, diagnosis, or therapy options for these people.

**Case presentation:**

On October 7, 2023, an 81-year-old man was admitted to the hospital primarily for recurrent chest tightness and pain that had persisted for more than 2 years and 1 month. Cerebral angiography upon admission revealed significant blockage of the right VA and stenosis of the left vertebral arterial origin. Six days following admission, a drug-eluting stenting procedure was carried out under local anesthesia to open the left VA origin via the femoral artery. Following the procedure, the patient experienced a progressive loss of muscle strength in all 4 limbs and paraplegia below the cervical 3 spinal cord. One week following the procedure, the patient was released from the hospital. After the procedure, the patient was released 1 week later. After the procedure, the patient’s symptoms persisted for a month.

**Conclusion:**

High awareness for high cervical cord infarction is required when neck discomfort and limb weakness with progressive progression arises after surgery. Complications of high cervical cord infarction following stenting for stenosis of VA origin are uncommon in clinical settings. Patients’ prognoses can be improved by prompt diagnosis and care.

## 1. Introduction

Spinal infarction is a rare disease of the nervous system, which causes the death of nerve cells due to spinal cord ischemia. Spinal infarction accounts for only 1% to 2% of all ischemic strokes and 5% to 8%^[1]^ of all acute myelopathy. In recent years, the incidence of ischemic cerebrovascular disease is increasing year by year, and tends to be younger. About 20% of ischemic strokes originate from the posterior circulation, and about 20% of posterior circulation strokes are associated with atherosclerotic vertebral stenosis,^[2]^ and about 9% to 33% of posterior circulation ischemia is caused by vertebral artery initiation stenosis (VAOS) or occlusion.^[3]^

Because of the relatively simple anatomical structure and vascular path of the vertebral artery initiation, interventional treatment is not complicated. Currently, the technology is becoming more mature and the success rate is high, so interventional treatment has become an important treatment measure for the stenosis of the vertebral artery initiation.^[4]^ However, the high cervical pulp infarction caused by initial vertebral artery stenting is very rare, and no detailed case reports have been reported in China so far.

## 2. Case presentation

This case study reports an 81-year-old man with “recurrent chest tightness and chest pain for 2 + years, followed by 1 month.” He was improved relevant examinations after admission on October 7: blood glucose was 9.4 mmol/L.24-hour holter electrocardiogram, the supracentricular early onset was 94/24H, and the right bundle branch block was incomplete. Routine color ultrasound: fatty liver; liver cyst; double kidney cyst; prostate calcification. CFP. Head computed tomography (CT): encephalomalacia foci in right occipital lobe, multiple lacunar foci in bilateral basal ganglia and thalamus, cerebral atrophy. Chest: Interstitial changes in both lungs. Multiple small nodules in both lungs. The carotid and vertebral artery color Doppler ultrasound indicated carotid artery plaque with stenosis. The national institute of health stroke scale (NIHSS) score is 0. No obvious abnormality was found in other laboratory and examinations tests. No obvious abnormality was found in neurological examination.

**Figure 1.**
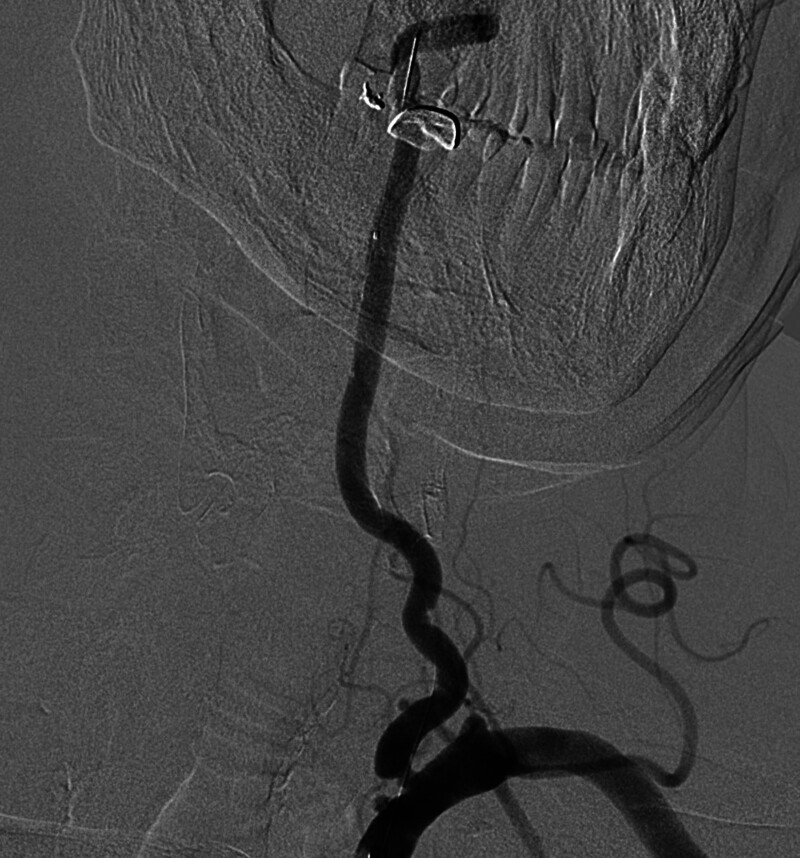
Cerebrovascular angiography diagnosis. 1. Left vertebral artery initiation severe stenosis, right vertebral artery occlusion, right posterior cerebral artery severe stenosis, occlusion? 2. Carotid plaque.

Coronary and cerebrovascular angiography was performed via radial artery on October 9. Coronary angiography diagnosis: coronary heart disease. The left anterior descending coronary artery is mildly stenosis, and the posterior descending coronary artery is moderately stenosis. Cerebrovascular angiography diagnosis: Left vertebral artery initiation severe stenosis, right vertebral artery occlusion, right posterior cerebral artery severe stenosis, occlusion? Carotid plaque (Fig. 1).

After communication with the family members, it was recommended to conduct vertebral artery stent surgery after 3 to 5 days of treatment with double adjuvant (aspirin 100 mg + clopidogrel 75 mg), and then continue to take diabetic low-salt and low-fat diet after surgery to improve circulation, reduce blood pressure, control sugar, anti-aggregation, stabilize plaque, and other treatments.

**Figure 2.**
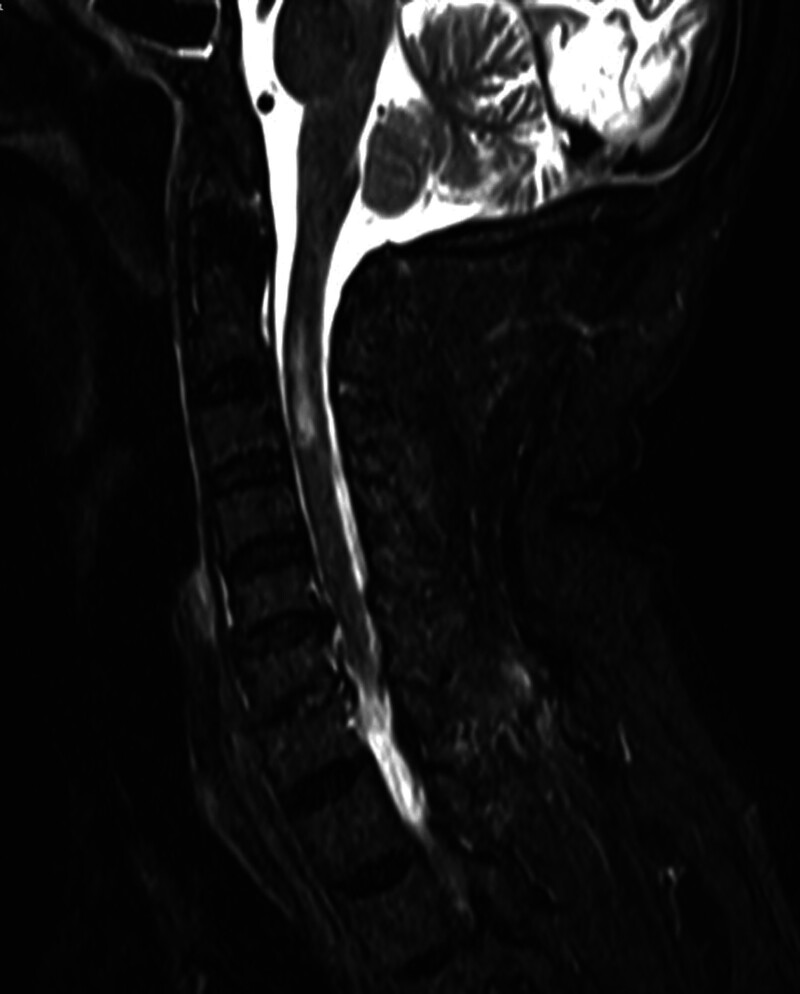
Relevant preoperative examination and preparation were improved, and after it was clear that there was no contraindication for surgery.

After relevant preoperative examination and preparation were improved, no contraindication was found for surgery. Percutaneous femoral artery drug-eluting stent implantation plus cerebrovascular angiography was performed under local anesthesia. The operation lasted 90 minutes. The operation was successful (Fig. 2). After the operation, nerve function recovery and fluid rehydration were given. The patient complained of neck pain and weakness in the left limb At 15: 30 on October 13.

Physical examination: left limb muscle strength level 3, rest of the nervous system physical examination was negative, NIHSS score 1 (upper limb movement L: 1, all others are 0 score). Consider the possibility of acute cerebral infarction, do not rule out the possibility of spinal cord infarction.

At 21:10, there is significantly increased weakness in the left limb, negative meningeal stimulation signs. Left limb muscle strength was level 1, left side mutual aid movement could not cooperate, right side was normal. NIHSS score 6 points (upper limb movement L: 3 points, lower limb movement L: 3 points, others were 0 score). The CTA examination of the head was improved, no obvious intracranial vascular abnormalities were found.

**Figure 3.**
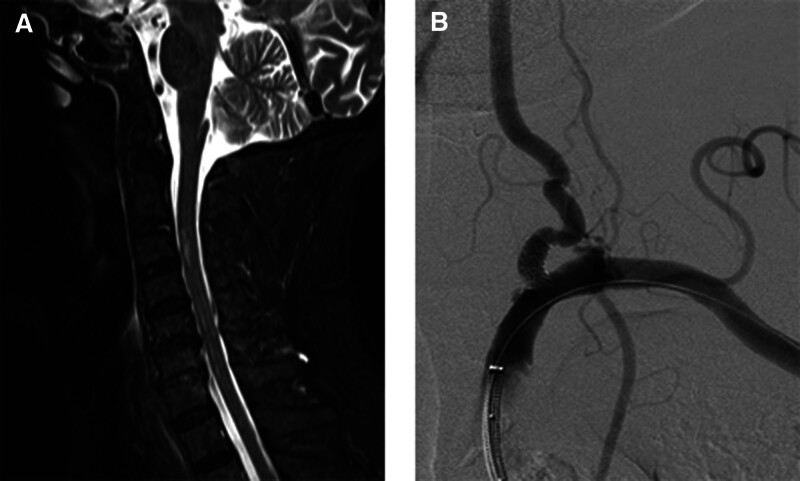
(A, B) 10 to 14 02:01 After continuing the communication with the family members, the emergency department underwent a DT examination of the head and MRI of the cervical vertebra again. A new acute cerebral infarction of the right cerebellum was considered after a radiographic review by a radiologist. T3 cervical infarction?/Cervical inflammation?

The emergency department underwent a CT examination of the head and magnetic resonance imaging (MRI) of the cervical vertebra again At 02:00 on October 14. A new acute cerebral infarction of the right cerebellum was considered after a radiographic review. T3 cervical infarction?/cervical inflammation? (Fig. 3A and B) The patient was treated with 100 mL of hormone saline + 240 mL of methylprednone sodium succinate, and the changes of limb movement, consciousness and vital signs were dynamically monitored.

At 17:30, the patient appeared to have speech lumps and his eyes stared upward. The possibility of aggravated stroke could not be ruled out. The patient was transferred to the intensive care unit for further treatment. Head CT showed that there were slightly more lacunar cerebral infarction than before, but the area of new cerebral infarction was not consistent with the patient’s signs.

After transfer to ICU, continue to take normal saline 100 mL + methylprednisolone sodium succinate 240 mg imvvqd, Antola acid inhibition to protect the stomach, Continue to use aspirin combined with hydropingrel to prevent platelet aggregation, papaverine hydrochloride + Nimodipine tablets to improve cerebral vasospasm, edaravone to improve cerebral blood flow, enoxaparin sodium 2000 U qd to prevent anticoagulation, atorvastatin calcium to regulate lipid, glycerin fructose to relieve brain tissue edema, sodium valproate to prevent epilepsy, and other symptomatic treatments.

**Figure 4.**
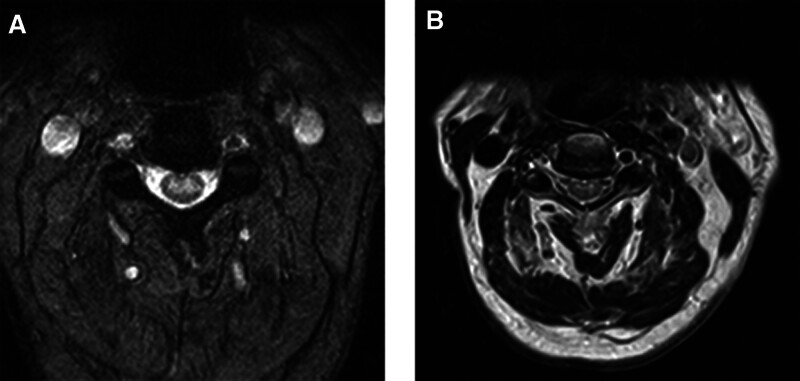
(A, B) October 15, 2023 The morning checkup reveals: clear consciousness, poor spirit, slightly pronounced, shortness of breath, left limb muscle strength grade 0, right upper limb muscle strength grade 2, right lower limb muscle strength grade 2, reexamination of the NMR reveals that the range of abnormal signals in the spinal cord at the T3 level has increased compared to October 14, 2023.

October 15, 2023: The morning checkup reveals: poor spirit, slightly pronounced, shortness of breath, left limb muscle strength grade 0, right upper limb muscle strength grade 2, right lower limb muscle strength grade 2, reexamination of the NMR reveals that the range of abnormal signals in the spinal cord at the T3 level has increased compared to October 14, 2023 (Fig. 4A and B).

The symptoms lasted for about 5 days and did not progress significantly. One week later, the family communicated with me and asked for automatic discharge.

Specialist physical examination at discharge: poor mental state, slurred speech, negative meningeal stimulation sign, left limb muscle strength level 0, Right limb muscle strength level 2, unable to cooperate with mutual aid movement. The improved rankin quantity scale scored 4 points.

Follow-up: the patient’s family refused to provide the patient’s recovery information, complaining of poor prognosis.

## 3. Discussion

The diagnosis of spinal infarction was clear. Spinal cord infarction is associated with many causes. These include vascular manipulation or variation associated with supplying the spinal cord (vertebral angiography, aortic or vertebral artery dissection), embolus formation (cardiogenic, fibrochondrogenic, large atherosclerotic), anatomic abnormalities (commonly subclavian artery), vasculitis (syphilis, autoimmune disease, primary central nervous system vasculitis), vascular malformations (commonly dural arteriovenous fistula), prone to coagulation (malignancy, anemia), and root artery compression from intervertebral discs and trauma. At present, according to relevant reports at home and abroad, the main cause of spinal cord infarction is atherosclerosis, and MRI is the most important examination method.^[5]^

The patient in this case had a clear history of stent implantation for severe stenosis at the onset of the vertebral artery before spinal infarction, which was considered as the cause of the infarction. Combined with MRI and clinical features, T3 anterior spinal artery (ASA) syndrome was considered as the diagnosis.

This article will analyze the causes of infarction from 3 aspects:

### 3.1. The anatomy of the vertebral arteries and the anatomy of the vertebral arteries after stenting

When congenital variation such as distortion and slender at the beginning of the vertebral artery occurs, it will increase the difficulty of intraoperative operation. In general, the left vertebral artery, which emanates from the aortic arch, is the left subclavian artery, the second grade branch of the aorta. The study found that there were many variations in the V1 segment of vertebral artery, which not only increased the difficulty of interventional operation, but also increased the risk.^[6,7]^ The vertebral artery originated from the autonomous arterial arch in 2.4%, originated from the anterior upper quadrant in 20.7%, and originated from the posterior upper quadrant in 79.3%.^[8]^

In this operation, the right vertebral artery was nondominant. The remodeling ability of the nondominant vertebral artery was lower than that of the dominant one, and the vertebral artery with small diameter was more likely to collapse under greater pressure. The nondominant side of the vertebral artery is more prone to atherosclerosis and vascular remodeling than the dominant side.^[9]^ Some domestic studies suggest that patients are more likely to have posterior circulation area infarction in nonmajor vertebral artery, which may be related to less blood rheology and slow blood flow rate in nonmajor vertebral artery, which are relatively more likely to form atherosclerotic plaques and thus lead to central infarction.^[10]^ This is also consistent with the diagnosis of right cerebellar and right occipital lobe infarction and right posterior cerebral artery P2 distal occlusion indicated by preoperative head CT and angiography in this patient.

In this case, the right vertebral artery was occluded, and the left vertebral artery was severely narrow and tortuous. During the operation, the 6F guide catheter was passed through the supra-aortic subclavian artery through the femoral artery, there were 2 times of guide catheter sliding events due to insufficient support of the guide catheter, and then it did not occur again by replacing the 8F guide catheter. Combined with Figures 1 and 2, it indicated that the stent implantation was difficult in this patient. The difficult anatomy of vertebral artery increases the risk of perioperative complications, such as dissection or tear, intimal injury, vasospasm, plaque displacement and so on.

Matula reported that the tortuosity rate of V1 segment of the vertebral artery was 47.2%, and the incidence of anatomical variation at the origin of the vertebral artery was high.^[11]^ A single surgical plan could not contain all the variations. Forcing stent implantation at the origin of the tortuous vertebral artery would increase vascular damage and eventually lead to plaque detachment or pseudodissection, resulting in thrombus detachment, and eventually induce infarction. Therefore, fully studying the vascular morphology is helpful to reduce the occurrence of complications and increase the benign prognosis rate of stent implantation.

Interventional surgery is a minimally invasive operation, non-non-invasive operation, stent as a foreign body, the process of implantation will inevitably cause different degrees of damage to the vessel wall, which leads to the body’s relative inflammatory healing response, the Journal of Brain and Neurological Diseases suggested that vascular elastic retraction and thrombosis usually occur in a few minutes to a few hours after interventional therapy. Neointimal hyperplasia and vascular remodeling typically develop within days to months after stenting. In fact, in the process of stent placement, the operation of catheter and guide wire, the release and expansion of stent, and the expansion of balloon will inevitably cause damage to the vessel wall and cause local inflammatory reaction. The pathological mechanism may be related to intimal hyperplasia, thrombosis, negative remodeling, and the imbalance between inflammatory and anti-inflammatory mediators.^[12–14]^ Inflammatory response is a normal physiological defense performance of the human body, but excessive inflammatory response may also lead to atherosclerosis or embolus formation and falling in the process of vascular remodeling, and the final outcome may be in-stent restenosis or distal cerebral infarction.

### 3.2. General conditions of patients

The prognosis of patients with ischemic stroke is related to their age and underlying diseases. Underlying diseases such as hypertension, hyperlipidemia, and diabetes are risk factors for ischemic stroke.^[15]^ In this case, the patient had received standardized dual antiplatelet therapy for 4 days before surgery, but the patient was older (>80 years), with multiple underlying diseases (coronary heart disease, multiple cardiac surgeries, hypertension and diabetes, hyperlipidemia, chronic renal insufficiency, right vertebral artery occlusion), and in poor general condition. It increases the risk of complications after stent implantation. Yan et al^[16]^ showed that serum low-density lipoprotein cholesterol (LDL-C), serum lipoprotein associated phospholipase A2 (Lp-PLA2), and E3/E4 genotype are independent risk factors for restenosis after stent implantation of vertebral artery origin segment. In addition, some studies have shown that bad living habits, antiplatelet drug resistance, irregular and irregular use of antiplatelet drugs can reduce the resistance of patients and increase the incidence of adverse cardiovascular and cerebrovascular events.^[17,18]^ Therefore, standardized use of therapeutic drugs, full evaluation of the general condition of patients, and control of indicators in the standard range can avoid the occurrence of surgical complications to the greatest extent.

Foreign studies have shown that contrast media have a certain degree of side effects on the nervous system, and the final morbidity rate is about 1% to 2%.^[19]^ Common manifestations include hemianopsia, epilepsy, hemiplegia, aphasia, cerebral hemorrhage, etc. Its incidence is as high as 4% after the use of hyperosmolar iodinated contrast media,^[20]^ and domestic scholars have found that it may be related to the spasm and hypoxia of tiny vessels supplying nerve cell tissue.^[21]^ However, the majority of contrast-induced injuries are contrast-induced encephalopathy, and there are no reports of relevant cases of contrast-induced spinal cord lesions. The clinical symptoms of contrast-induced encephalopathy usually occur within minutes to days after intravenous infusion of contrast medium, and disappear within about 3 days, and most of them have a good prognosis. In this case, the patient was aged and had renal insufficiency. Iodixanol was selected as the contrast agent. The surgeon had diluted the contrast agent to a considerable extent before operation.

### 3.3. Anatomical factors of the spinal cord

The blood supply of the spinal cord is mainly from the ASA, posterior spinal artery (PSA) and root artery. In this case, the patient was diagnosed with T3 infarction in the upper cervical spinal cord, and emboli embolism in the vertebral artery supply area was considered, which was related to stent surgery. A multicenter retrospective study by Adam et al of institutions using flow diverter device (FD) for the treatment of vertebral artery (VA) aneurysms from 2011 to 2019 assessed the risk of ASA and PSA/lateral spinal artery (LSA) occlusion, associated thromboembolic complications, overall complications, and functional outcome. Treatment of posterior circulation FD aneurysms covered by ASA or PSA/LSA was found not to be associated with these branch occlusion rates or with any cases of spinal cord infarction.^[22]^

The treatment of superficial vertebral artery dissecting aneurysm with FD device is relatively safe at present, which has certain reference significance for the later use of FD device to prevent embolization complications caused by stent implantation in difficult anatomical vertebral artery stenosis. The treatment of superficial vertebral artery dissecting aneurysm with FD device is relatively safe at present, which has certain reference significance for the later use of FD device to prevent embolization complications caused by stent implantation in difficult anatomical vertebral artery stenosis.

### 3.4. Surgical and intraoperative decision-making factors

Surgical factors include intraoperative strategy, operator’s operating skills, selection of operating methods, intraoperative timing, etc. Among them, the author believes that the intraoperative operation is the main cause and is the direct determinant factor affecting the prognosis. Studies have shown that insufficient stent expansion and malapposition during the procedure are clinically associated with poor outcomes after vertebral artery stenting.^[23]^ As mentioned above, the 6F guide catheter was not enough support during the operation, so the 8F guide catheter needed to be selected to strengthen the support. At the same time, the double micro-guide wire technology was used to place the loach guide wire into the distal subclavian artery to maintain stability. This demonstrates the flexible application of intraoperative strategy, operation method and timing, and the final successful completion of the operation. The operation and follow-up of 15 patients who underwent stent angioplasty for vertebral artery stenosis through the radial approach showed that this approach may also be a relatively safe and reliable method.^[24]^ Domestic studies have recommended that when the Angle between the V1 segment of the vertebral artery and the horizontal segment of the subclavian artery is >90°, it can be used as a reference condition for stent angioplasty of vertebral artery stenosis via the radial artery approach.^[25]^ In this case, it was difficult to perform stent angioplasty of vertebral artery ostial stenosis through femoral artery approach. Before operation, the radial artery approach was considered, but it was found that the initial segment was tortuous and narrow after calculation, and there was still a probability of insufficient catheter support during operation, so this approach was given up. Joshi et al in the UK searched 3 online databases (MedLine via PubMed, Scopus and Embase) and concluded that radial artery access (TRA) has a low complication and recurrence rate for interventional procedures and treatment. With the increasing maturity and development of TRA-specific neurovascular devices, it is safe and feasible to use radial artery approach for interventional procedures and treatment. Along with continued reports of its success in the literature, TRA is expected to be more widely used among neurointerventionalists.^[26]^ In addition, vertebral endarterectomy is less preferred by patients and neurosurgeons as the surgical treatment of vertebral artery stenosis due to its large trauma, long postoperative recovery period, more postoperative complications, and higher requirements for anesthesia tolerance than interventional stenting. A Shandong University study evaluating the safety and effectiveness of microsurgical reconstruction of the proximal VA surface, Microsurgical reconstruction is an alternative option for effective treatment of refractory proximal VASO disease and in-stent stenosis, with a high rate of postoperative vascular reciraturation.^[27]^ This suggests that the operator may try the transradial approach or vertebral endarterectomy under appropriate circumstances as an option when stent angioplasty of vertebral artery ostial stenosis via femoral artery approach is difficult.

#### 3.4.1. Intraoperative stent type and size

At present, the common types of vertebral artery stents include balloon-expanded stents, drug-coated stents, and bare-metal stents. In contrast, drug-coated stents have anti-inflammatory and antiintimal proliferative effects that prevent thrombosis and prevent vascular remodeling.^[28]^ A meta-analysis by Tank et al found significantly lower rates of restenosis, symptomatic recurrence, and in-stent restenosis in the drug-coated balloon stent (DCB) group compared with the bare-metal stent (BMS) group.^[29]^ In their study comparing the number of emboli shed during percutaneous transluminal angioplasty/stenting of the vertebral artery (VA) with the number released during stenting of the internal carotid artery (ICA), Divani et al^[30]^ showed that the frequency and number of emboli captured during stenting procedures of the ICA and VA were comparable; therefore, The use of a distal umbrella device (EPD) for stenting in the vertebral artery is recommended. In this case, EPD was placed in the distal V2 of the left vertebral artery at the active request of the patient’s family due to good preoperative communication and fully informed of the cost and risk of the operation. A comparison of the angiographic and clinical outcomes of drug DCB with distal embolic protection device (EPD) versus BMS without EPD for symptomatic vertebral artery originating stenosis (VAOS) also showed that DCB combined with EPD was technically feasible and safe for symptomatic VAOS compared with BMS without EPD. There was also a significant reduction in radiographic thromboembolic events.^[31]^ This indicates that the preoperative consideration of the surgeon is sufficient. In the case of unpredictable outcome of spinal cord infarction, we have not found an effective way to further consider the prevention of small artery embolization.

In addition, the size and selection of stent will further affect the success rate of implantation and the incidence of complications. Compared with the normal artery cross section, the stent with a larger diameter has a higher stimulation of the blood vessel and induces a more severe inflammatory response. The stent with a smaller diameter may lead to surgical failure or an increased risk of embolism. Fukuda et al^[32]^ proposed a vascular reconstruction technique using computed tomography angiography (CBCT-A) to evaluate the detailed vascular system and the relationship between vertebral artery opening, dissection, and surrounding vessels. In the experiment, CBCT-A clearly showed the luminal morphology of the intimal valve/double lumen, the entrance of the false lumen, and the entire anatomical segment. Small perforator arteries were also found, and the detailed anatomical results obtained by using CBCT-A will help to obtain safe and effective treatment outcomes. During this operation, Maurora drug-coated stent was used, and the maximum diameter of the left vertebral artery was measured to be 4.8 mm, which was implanted at 4.5 × 12 mm. Due to the occlusion of the right vertebral artery and the lack of blood supply of the ASA, the possibility of the stent covering the tiny perforator artery for the blood supply of the ASA could not be excluded under the condition of unclear conventional angiography. Finally, it resulted in T3 cervical spinal cord infarction. The study by Fukuda et al reveals a new approach in which, with a better understanding of the anatomy of the vertebral artery and peripheral blood supply, selection of a more appropriate stent size and size can provide adequate protection and support for a positive prognosis.

## 4. Conclusions

In conclusion, high cervical spinal cord infarction after stent implantation of vertebral artery origin stenosis is still rare in clinic. When the patient has neck pain, limb weakness, and progressive symptoms after vertebral artery stenting, the possibility of high cervical spinal cord infarction should be highly vigilant. Good anatomical knowledge, skilled and accurate operation, correct selection of surgical methods and intraoperative judgment, accurate evaluation of surgical indications and perfect surgical plan can avoid the occurrence of complications after vertebral artery intervention to the greatest extent and improve the prognosis of patients.

## Author contributions

**Writing – original draft:** Wen Cheng, Jiangbin Wu.

**Writing – review & editing:** Quanlong Yang, Xiaodong Yuan.
